# Can Domestic Cats Act as Reservoirs of *Cytauxzoon felis*?

**DOI:** 10.3390/pathogens12020266

**Published:** 2023-02-06

**Authors:** Adrian Alberto Díaz-Sánchez, Alejandro Cabezas-Cruz

**Affiliations:** 1 Department of Biology, University of Saskatchewan, 112 Science Place, Saskatoon, SK S7N 5E2, Canada; 2 ANSES, INRAE, Ecole Nationale Vétérinaire d’Alfort, UMR BIPAR, Laboratoire de Santé Animale, F-94700 Maisons-Alfort, France

Cytauxzoonosis is a worldwide tick-borne disease of domestic and wild felids, caused by infection of the haemoparasites belonging to the genus *Cytauxzoon* (Piroplasmida, Apicomplexa)*,* which are apicomplexan protozoans closely related to those of the genera *Babesia* and *Theileria* [1]. In this group of pathogens, *Cytauxzoon felis* is the most prominent species that was first described by Wagner [2] in the mid-1970s in domestic cats from Missouri, United States. The biological transmission to wild and domestic felids occurs mainly by tick vectors *Amblyomma americanum* and *Dermacentor variabilis*, but recent studies defined *A. americanum* as the primary definitive host and tick vector of *C. felis* [3,4]. Originally, it was hypothesized that this parasite occurs only in the Nearctic Region, mainly in the United States, where bobcats (*Lynx rufus*) have been described to serve as the main natural reservoir host with clinically self-limited infections, usually remaining asymptomatic [5]. However, in the beginning of the XXI century, *Cytauxzoon* spp. infections with a species molecularly different to *C. felis* were described in wild and domestic felids in Europe [6], Eurasia [7], and South America [8]. In Europe, *Cytauxzoon* spp. was reported primarily in southern and later in central Europe, where it was detected in all three species of European wild felids including European wildcats (*Felis silvestris*) [9], Iberian lynx (*Lynx pardinus*) [6], and Eurasian lynx (*Lynx lynx*) [10], as well as in domestic cats (*Felis catus*) [11,12]. In Europe, the *Cytauxzoon* spp. infection is gaining clinical importance as it is being considered a newly emerging tick-borne disease in felids [13]. The Eurasian and the Iberian lynx play the role of natural reservoirs of *Cytauxzoon* spp., similarly to bobcats who serve as the primary reservoir of *C. felis* in the United States [5], while the competent tick vector involved in the biological transmission of this piroplasm in Europe has not yet been identified.

Despite recent advances in understanding the epidemiology of *C. felis,* a major question remains as to whether domestic cats can act as reservoirs for this pathogen. One of the Editor’s choice articles in 2020 published in *Pathogens* by Wikander et al. [14] reported two important findings shedding new light into the role of domestic cats in the epidemiology of *C. felis* in United States. Firstly, these authors found that a *C. felis* infection in domestic cats is more prevalent than previously thought. Secondly, they found that many infected cats survived the acute phase of the disease. Based on these findings, the authors concluded that persistently parasitemic individuals could act as a disease reservoir for other felids. Keeping up to date, several *Cytauxzoon* spp. have been identified in felids from Europe and North America, where *C. felis* is still considered to be the most important with an expanding distribution in the United States [15]. Historically, infection with *C. felis* in domestic cats is described as a highly fatal disease distinguished by an acute or peracute severe clinical syndrome with a wide spectrum of clinical signs such as pyrexia, anorexia, lethargy, depression, dehydration, dyspnea, hemolytic crisis, icterus, and hepatosplenomegaly [16,17]. The evidence now reported by Wikander et al. [14] adds to previous studies [18,19,20], showing that domestic cats can recover from acute cytauxzoonosis, which often remains chronically infected for a long period of time and serves as a potential reservoir of the parasite for subsequent tick transmission to other species of domestic, wild, and captive felines [18,20]. In fact, in the last two decades, several cross-sectional studies confirmed by PCR and DNA sequencing found the occurrence of *C. felis* to be nonfatal and asymptomatic in healthy domestic cats from enzootic areas of the southern and southeastern United States [19,20]. Whether domestic cats could also act as reservoirs of *Cytauxzoon* spp. in Europe remains a question.

In modern times, the phylogenetic studies based on 18S rDNA gene sequences have showed that *Cytauxzoon* spp. isolates from Europe are phylogenetically related but distinct from *C. felis* isolates in North America [10,11]. The *Cytauxzoon* spp. isolates from North America and Europe form two separate subclades into the *Cytauxzoon* monophyletic group, with a low inner diversity of the European clade [21]. Interestingly, European *Cytauxzoon* spp. isolates are most closely related to *Cytauxzoon manul* described in the Pallas’ cats (*Otocolobus manul*) from Mongolia [22,23]. Recently, new molecular evidence described by Panait et al. [24] based on the phylogenetic analyses of two mitochondrial genes, cytochrome *b* (*CYTB*) and cytochrome *c* oxidase subunit I (*COI*), suggested that three new species of *Cytauxzoon* occur in European wild felids, which were named as *Cytauxzoon europaeus*, *Cytauxzoon otrantorum*, and *Cytauxzoon banethi*. Currently, only *C. europaeus* infections have been reported in domestic cats [25], while whether the other two new *Cytauxzoon* sp. species also occur in domestic cats remains unknown, since data on the European isolates of *Cytauxzoon* spp. are still scarce.

Over the past 40 years, knowledge about the feline cytauxzoonosis and its causal agent *C. felis* has grown considerably. However, it is surprising that despite the importance of cytauxzoonosis for domestic cats described in previous studies, this disease remains scarcely studied, even among veterinarians. In fact, to date, only 221 research articles on “*Cytauxzoon*” have been published (PubMed, https://pubmed.ncbi.nlm.nih.gov/, accessed on 1 January 2023), that contrast with thousands of articles dealing with other apicomplexan parasites transmitted by ticks such as *Babesia* and *Theileria*. Thus far, there are no commercially available diagnostic kits and vaccines for detection and prevention of *C. felis* infections, respectively. The most effective method to prevent disease in domestic cats is restricted to prophylactic ectoparasite control and limiting outdoor exposure, insomuch as ticks are the only described mechanisms of transmission. Therefore, since *C. felis* is considered to be a new emerging tick-borne pathogen, understanding the role of domestic cats in the biology, ecology, epidemiology, and clinical aspects of feline cytauxzoonosis warrant further research focus on developing disease mitigation strategies.
